# Forkhead transcription factor Fkh1: insights into functional regulatory domains crucial for recruitment of Sin3 histone deacetylase complex

**DOI:** 10.1007/s00294-021-01158-3

**Published:** 2021-02-26

**Authors:** Rasha Aref, Marwa N. M. E. Sanad, Hans-Joachim Schüller

**Affiliations:** 1 Department of Genetics, Faculty of Agriculture, Ain Shams University, Shoubra El-Khaymah, Cairo, 11241 Egypt; 2 Department of Genetics and Cytology, National Research Centre, Cairo, Dokki Egypt; 3 Center for Functional Genomics of Microbes, Abteilung Molekulare Genetik Und Infektionsbiologie, Felix-Hausdorff-Straße 8, 17487 Greifswald, Germany

**Keywords:** Histone deacetylases (HDACs), Fkh1, Sin3, Tup1, Cell cycle genes

## Abstract

Transcription factors are inextricably linked with histone deacetylases leading to compact chromatin. The Forkhead transcription factor Fkh1 is mainly a negative transcriptional regulator which affects cell cycle control, silencing of mating-type cassettes and induction of pseudohyphal growth in the yeast *Saccharomyces cerevisiae*. Markedly, Fkh1 impinges chromatin architecture by recruiting large regulatory complexes. Implication of Fkh1 with transcriptional corepressor complexes remains largely unexplored. In this work we show that Fkh1 directly recruits corepressors Sin3 and Tup1 (but not Cyc8), providing evidence for its influence on epigenetic regulation. We also identified the specific domain of Fkh1 mediating Sin3 recruitment and substantiated that amino acids 51–125 of Fkh1 bind PAH2 of Sin3. Importantly, this part of Fkh1 overlaps with its Forkhead-associated domain (FHA). To analyse this domain in more detail, selected amino acids were replaced by alanine, revealing that hydrophobic amino acids L74 and I78 are important for Fkh1-Sin3 binding. In addition, we could prove Fkh1 recruitment to promoters of cell cycle genes *CLB2* and *SWI5.* Notably, Sin3 is also recruited to these promoters but only in the presence of functional Fkh1. Our results disclose that recruitment of Sin3 to Fkh1 requires precisely positioned Fkh1/Sin3 binding sites which provide an extended view on the genetic control of cell cycle genes *CLB2 and SWI5* and the mechanism of transcriptional repression by modulation of chromatin architecture at the G2/M transition.

**Supplementary Information:**

The online version contains supplementary material available at 10.1007/s00294-021-01158-3.

## Introduction

The orchestrated action between transcription factors and regulatory corepressor complexes governs the chromatin organization and thereby gene expression. A central question in the field of epigenetics is how functional protein complexes can be correctly recruited by transcription factors and decipher the encoded protein networks that dictate cellular function. The family of forkhead box transcription factors is conserved from yeasts to mammalian cells and represents a crucial class of transcriptional regulators, affecting miscellaneous functions such as cell cycle control, differentiation, DNA repair, apoptosis, oxidative stress and autophagy (van der Horst and Burgering 2007; Hannenhalli and Kaestner 2009; Storz 2011; Postnikoff et al. 2012). In multicellular systems, dysfunction of forkhead box subfamily O regulators (FoxO) was revealed as a driving force of cancer progression and tumorigenesis (reviewed in Farhan et al. 2017). The genome of the yeast *S. cerevisiae* encodes four Fox proteins among which Fkh1 and Fkh2 (Forkhead homolog) are important regulators of the *CLB2* cluster of cell cycle-regulated genes, responsible for the transition of the G2/M boundary (Jorgensen and Tyers 2000; Murakami et al. 2010). Fox proteins contain a forkhead box with a winged-helix structural motif as a DNA-binding domain together with a ForkHead Associated (FHA) domain which is required for protein–protein-interactions with an emphasis on recognition of phosphopeptides (Durocher and Jackson 2002; Dummer et al. 2016). In *S. cerevisiae, FKH1* was identified as a gene which increases silencing of the mating-type cassette *HMRa* but also affects pseudohyphal growth (Hollenhorst et al. 2000) and firing of origins of DNA replication (Reinapae et al. 2017). Characterization of *fkh* single and double mutants revealed that *FHK1* and *FKH2* may have redundant roles but can also execute distinct functions (Hollenhorst et al. 2000; Zhu et al. 2000), depending on the genetic context. Importantly, Fkh2 but not Fkh1 has been identified as a subunit of the Mcm1-containing activator complex SFF (Swi five factor), responsible for regulated expression of *SWI5* and *CLB2* in G2 of the yeast cell cycle (Pic et al. 2000).

Forkhead transcription factors may control gene transcription by recruiting coactivators and/or corepressors, leading to coordinate activation and/or silencing of genes (Lalmansingh et al. 2012). In particular, transcriptional repression by corepressors can be executed by inhibiting the basal transcription machinery or by on-site recruitment of chromatin remodeling activities (Burke and Baniahmad 2000). In yeast, two transcriptional corepressor complexes namely Sin3/Rpd3 and Cyc8/Tup1 (Silverstein and Ekwall 2005; Malave´ and Dent 2006; Váchová and Palková 2019) mediate gene repression by interacting with DNA-binding factors, thereby recruiting histone deacetylases to the target promoters which leads to compact chromatin. Sin3 is devoid of any enzymatic activity but functions as a scaffold protein, using its four paired amphipathic helix motifs (PAH1-PAH4; Wang et al. 1990) to establish various protein–protein interactions. A similar function is fulfilled by ten tetratricopeptide repeat motifs (TPR, each consisting of 34 amino acids) found at the N-terminus of Cyc8 and by WD40 repeats at the C-terminus of Tup1 (Malave´ and Dent 2006). Interestingly, a single domain of repressor Opi1 is able to interact with PAH1 of Sin3 and with TPR motifs of Cyc8 (Jäschke et al. 2011). Interaction of Fkh1 with corepressor Sin3 was initially uncovered by high-throughput mass spectrometric protein complex identification (HMS-PCI; Ho et al. 2002). Fkh2 is able to recruit Sin3/Rpd3 for repression of the *CLB2* gene cluster Reynolds et al. 2003; Veis et al. 2007), while its phosphorylation-dependent activation requires Fkh2 and not Fkh1 with coactivator Ndd1 associated by Clb/Cdk1 activities (Linke et al. 2017; Shi 2016).

No details of the interaction between Fkh1 and Sin3 were previously investigated. Since repressor proteins are able to contact several corepressors (Jäschke et al. 2011; Aref and Schüller 2020) we thus studied whether Fkh1 can also bind to Cyc8 and Tup1. In this report, we show that Fkh1 interacts directly with both Sin3 and Tup1 corepressors but not with Cyc8. We also identify the interaction domain of Fkh1 responsible for binding to Sin3. Mutational studies showed that hydrophobic amino acids L74 and I78 are important for Fkh1-Sin3 binding. Chromatin immunoprecipitation (ChIP) analyses ascertained Fkh1 recruitment to cell cycle-regulated promoters *CLB2* and *SWI5*. Importantly, Sin3 was also recruited to these promoters but only in the presence of functional Fkh1.

## Material and methods

### Yeast strains and media

For ChIP analyses, derivatives of *S. cerevisiae* haploid strain C13-ABY.S86 lacking four vacuolar proteinases (*pra1 prb1 prc1 cps1*; De Antoni and Gallwitz 2000) were used. An epitope-tagged variant of *FKH1* at its authentic chromosomal position was introduced by gene replacement (construction of epitope-tagged variant of *FKH1* at its authentic chromosomal positions; see below). Complete genotypes of all used strains are available in the supplementary file, Table 1 (section a).

### Plasmid constructions

To perform interaction assays, *Escherichia coli* expression plasmids (derived from pGEX-2TK; GE Healthcare) encoding various glutathione *S*-transferase (GST) fusions were constructed. Length variants of coding regions of the *FKH1* gene were amplified by PCR and fused behind GST. Similarly, HA-tagged length variants of Sin3 representing PAH domains, Cyc8 and Tup1 were expressed in yeast using plasmid p426-MET25HA (Mumberg et al. 1994). For bacterial expression of selected Sin3 variants, plasmid pASK-IBA5 (tetR-regulated; IBA, Göttingen, Germany) was used. Yeast expression plasmid pCW117 used for the synthesis of HA_3_-tagged Sin3 (full-length) has been described (Wagner et al. 2001). For the bacterial synthesis of epitope-tagged Sin3, Cyc8 and Tup1 plasmids pSW11 (HA_3_-*SIN3*; full-length), pFK77 (HA_3_-*CYC8*; encoding aa 1–398 representing the TPR-containing domain) and pRAR110 (HA_3_-*TUP1*; full-length) derived from pASK-IBA5 (tetR-dependent; IBA, Göttingen, Germany) were used. To confirm authenticity of gene fragments obtained by PCR, GST fusions which encode minimal length variants of Fkh1 were verified by DNA sequencing (LGC Genomics, Berlin, Germany). Plasmid names and fused sequences are mentioned in legends of figures and are described in detail in supplementary file Table 1 (section b). Gene-specific primers used for PCR amplifications are available in supplementary file Table 1 (section c). Plasmid pRAR107 was constructed by established procedures to disrupt the *FKH1* gene (*Δfkh1::LEU2*). To construct this plasmid, flanking sequences upstream and downstream of the respective coding region were amplified by PCR and inserted on both sides of *LEU2* selection marker, allowing total deletion of the *FKH1* reading frame.

### In vitro interaction assays (GST pull-down)

GST- and HA-tagged proteins used for interaction assays by affinity chromatography were synthesized by *E. coli* strain BL21 (Stratagene/Agilent). The tac promoter controlling GST fusion genes was induced with 1 mM IPTG. Similarly, tetR-dependent gene expression was activated by 0.2 mg/l anhydrotetracycline. Derepression of *MET25-*dependent gene fusions was achieved by cultivating yeast transformants in the absence of methionine. GST fusion proteins synthesized in *E. coli* were released by sonication, immobilized on glutathione (GSH) sepharose and subsequently incubated with yeast or bacterial total protein extracts containing HA fusions. To avoid unspecific interactions, protein extracts were pre-cleared by treatment with GSH Sepharose beads prior to incubation with GST fusions. After the release of GST fusions with free GSH (10 mM), eluates were separated by SDS-PAGE and proteins transferred to a filter. Following incubation with anti-HA-peroxidase conjugate, HA fusion proteins were detected with POD chemiluminescent substrate (antibody conjugate and substrate from Roche Biochemicals).

### Two-hybrid assays

To perform two-hybrid assays, strain PJ69-4A was used (*MATa trp1-901 leu2-3,112 ura3*-*52 his3*-*200 gal4*Δ* gal80*Δ* UAS*_*GAL2*_-*ADE2 LYS2*::*UAS*_*GAL1*_-*HIS3 met2*::*UAS*_*GAL7*_-*lacZ*; James et al. 1996). DNA fragments encoding interaction domains of Sin3 (PAH1 & PAH2) were inserted into plasmids pGBD-C1 (2 µm GAL4_BD_ *TRP1*) while Fkh1 domains (aa 1–125; aa 126–240; aa 81–160; aa 51–125) were inserted in pGAD-C1 (2 µm GAL4_AD_ *LEU2*). Double-transformed strains containing both types of fusion plasmids were first selected on a medium lacking leucine and tryptophan (-L-T) and subsequently transferred to a medium devoid of adenine (-L-T-A).

### Site-directed mutagenesis

To alter selected residues in the coding region of Fkh1, the QuikChange site-directed mutagenesis kit of Stratagene was used. To obtain mutations within the FHA domain, plasmid pRAR73 containing the Fkh1 coding region was used. To replace selected residues against alanine, we used pairs of mutagenic primers introducing a GCA codon instead of the natural codon, flanked by 15–19 nucleotides on both sides. DNA sequencing was used to confirm the presence of the desired mutant alleles of *fkh1* (L74A and I78A) and the absence of any other change in the plasmids obtained (pRAR89, pRAR90).

### Chromatin immunoprecipitation

Chromatin immunoprecipitation (ChIP) analysis followed the procedure described by Cobb and Van Attikum (2010). Chromosomal locus *FKH1* was modified such that it expressed a His-tagged Fkh1 without alteration of gene copy number or control region. Tagging was performed by the transformation of strain C13-ABY.S86 with a gene-specific modification fragment and selection for resistance against geneticin. The modification fragment was amplified by PCR, using gene-specific primers and plasmid pU6H3HA as a template (contains a His_6_-HA_3_-kanMX cassette; De Antoni and Gallwitz 2000). A strain which encodes epitope-tagged Sin3 (FKY11) was kindly provided by F. Kliewe. To introduce an *fkh1* gene deletion, strain FKY11 was transformed with the gene disruption cassette from plasmid pRAR107. The resulting strains RAY4 (*FKH1-HIS*_*6*_*-HA*_*3*_*-kanMX*), FKH11 (*SIN3-HIS*_*6*_*-HA*_*3*_*-kanMX*) and its isogenic *fkh1* derivative RAY5 (Δ*fkh1 SIN3-HIS*_*6*_*-HA*_*3*_*-kanMX)* grew until mid-log phase and were treated with formaldehyde for 15 min.

The crosslinking reaction was subsequently quenched for 5 min by the addition of glycine to a final concentration of 125 mM. After lysis, cells were sonicated five times for 30 s to shear chromatin, using a Bandelin Sonoplus UW 70 microtip (35% power). After sonication, lysates were centrifuged for 10 min at 16,000 *g* to remove insoluble material and incubated for at least 4 h with His-Tag Dynabeads^®^ (Invitrogen/Dynal^®^). After elution of affinity-purified proteins and bound DNA with a buffer containing 300 mM imidazole, cross-linking was reversed by heating to 65 °C overnight. DNA was recovered and analyzed by PCR (29 amplification cycles), using specific primers for promoters of *CLB2* (− 880/− 580), *SWI5* (− 420/− 170) or *ACT1* (+ 841/ + 1165, negative control).

### In silico analysis

Based on the methods of homology, the secondary structure of Fkh1 protein was predicted using an accurate web-based predicting tool Phyre2 (Kelley et al. 2015) and was reanalyzed in reference to similar secondary structures using Jpred tool (Cole et al. 2008, and based on JNet method that uses various neural networks. The tertiary structure (3D) was predicted in complementary to the secondary structure using Phyre2, modeled and visualized using JSmol (Hanson et al. 2013).

### Reporter gene assay

Effector plasmid encoding Fkh1 (full length) fused with the DNA binding domain of lexA (supplementary Table 1b) was transformed into two strains containing integrated reporter genes (*CYC1-lacZ* without lexA-binding site; *CYC1-lacZ* with four upstream lexA-binding sites), selecting on SCD -Ura -Leu medium. Transformants were cultured in double-selective medium at 30 °C until mid-logarithmic growth phase and then specific β-galactosidase activities were determined in the crude extract. The empty repressor test plasmid pRT-lexA was used as a control.

### Miscellaneous procedures

Transformation of *S. cerevisiae* strains, selection for yeast transformants on the respective synthetic media, PCR amplification and β-galactosidase assays have been described (Schwank et al. 1995; Wagner et al. 2001).

## Results

### Protein interaction network of Fkh1 and Sin3 co-repressor association

**Fig. 1:**
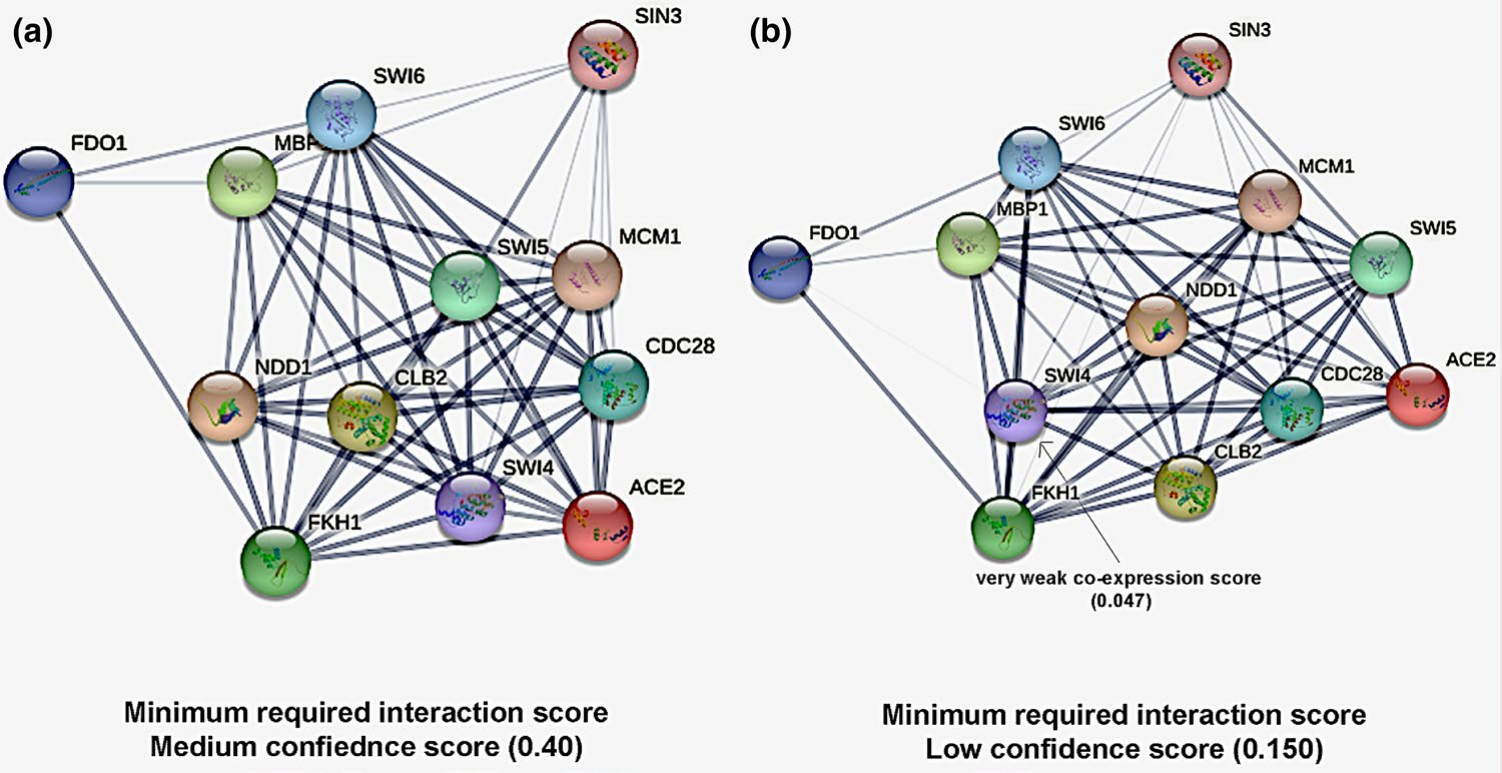
The known full interaction network of Fkh1 in addition to Sin3 protein. According to the current status in STRING database, Fkh1/Sin3 interaction is uncharacterized (green line) in *S. cerevisiae* or other organisms on the medium confidence level of 0.4 (**a**). At a low confidence score of 0.150 no interaction in *S. cerevisiae* and very weak co-expression score (0.047) and a low combined score (0.37) was detected in putative homologs in other organisms (**b**)

To explore the interaction between Fkh1 and Sin3, it was essential to identify the known and current full functional and physical interaction network of Fkh1 in the presence of Sin3. The information was retrieved from the PPI database of STRING (Szkalrczyk et al. 2019). The interaction between Fkh1 and Sin3 is uncharacterized in *S. cerevisiae* or other organisms at a medium confidence level of 0.4 interaction scores (Fig. 1a). Also, at a low confidence interaction score (0.150) (Fig. 1b), no interaction was detected in *S. cerevisiae,* while a very weak co-expression of 0.047 was found between Fkh1/Sin3 of the putative homologs of Fkh1 and Sin3 in other organisms (Xiong et al. 2014).

### Fkh1 directly binds to the pleiotropic corepressor Sin3

**Fig. 2:**
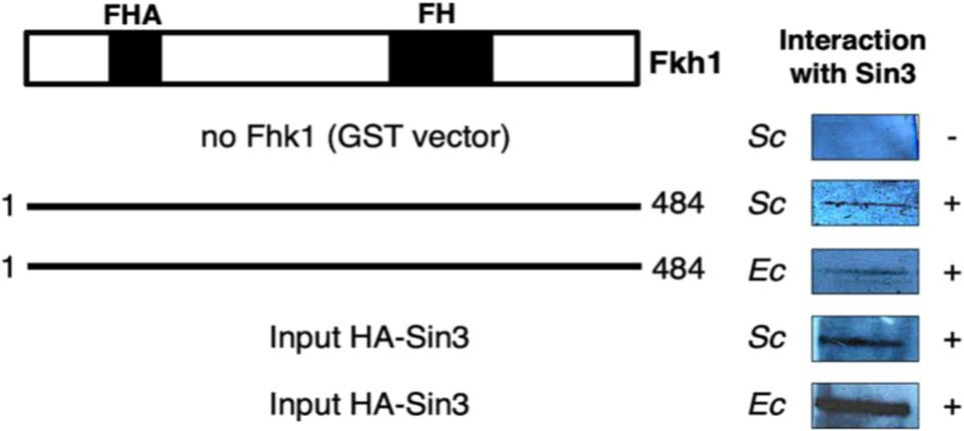
In vitro interaction of Fkh1 and Sin3 shown by affinity chromatography. GST-Fkh1 (full-length fusion protein, 1–484) bound to glutathione (GSH) sepharose was incubated with protein extract containing epitope-tagged Sin3 (full-length) expressed in *S. cerevisiae* (*Sc*; plasmid pCW117) and in *E. coli* (*Ec*; pSW11) separately. GST-Fkh1 fusion protein was released from the affinity matrix together with its partner by free GSH and subsequently separated by SDS-PAGE, followed by immunodetection using an anti-HA antibody. GST vector was used as a negative control. Input controls are shown at the bottom of Fig. (20% of protein used for the interaction assay). *FH* forkhead domain, *FHA* forkhead associated

A high-throughput characterization of yeast protein complexes by mass spectrometry provided evidence for a physical interaction of Fkh1 with corepressor Sin3 (Ho et al. 2002). This finding prompted us to use affinity chromatography for demonstrating Fkh1 interaction with Sin3 in vitro. A glutathione-*S*-transferase (GST)-Fkh1 fusion protein (amino acids 1–484, comprising full length) was synthesized in *E. coli* and subsequently bound to glutathione (GSH) sepharose. A protein extract from *S. cerevisiae* transformants containing epitope-tagged Sin3 (full length) was added to this affinity matrix. After intensive washing with increasing stringency, specifically bound protein was eluted by the addition of free GSH. As is shown in Fig. 2, immunodetection with anti-HA-antibody confirmed that the 175 kDa protein HA_3_-Sin3 could be bound by GST-Fkh1 under stringent conditions but not by GST. Thus, Fkh1 may execute its function by recruiting the general corepressor Sin3.

As interaction experiments performed with protein extracts from yeast cannot completely rule out indirect interactions mediated by distinct factors, HA-Sin3 was thereafter also synthesized in *E. coli*. Since bacterial protein extracts should not contain yeast-specific factors, a direct interaction can be concluded by the use of HA-Sin3 from *E. coli*. As is apparent from Fig. 2, identical results were indeed obtained with extracts from *E. coli*, indicating that interaction between Fkh1 and Sin3 occurs directly.

### Domains mediating physical interaction of Fkh1 and Sin3

To define more precisely the indispensable parts responsible for Fkh1 binding to Sin3, GST fusions of Fkh1 length variants were immobilized and subsequently incubated with HA-Sin3 length variants from yeast. Wang and Stillman (1993) proposed four PAH motifs in Sin3 required to mediate various protein–protein interactions. Therefore, HA-Sin3 length variants representing individual structural and functional domains (PAH1-PAH4, HID) were tested in the pull-down assay.

**Fig. 3:**
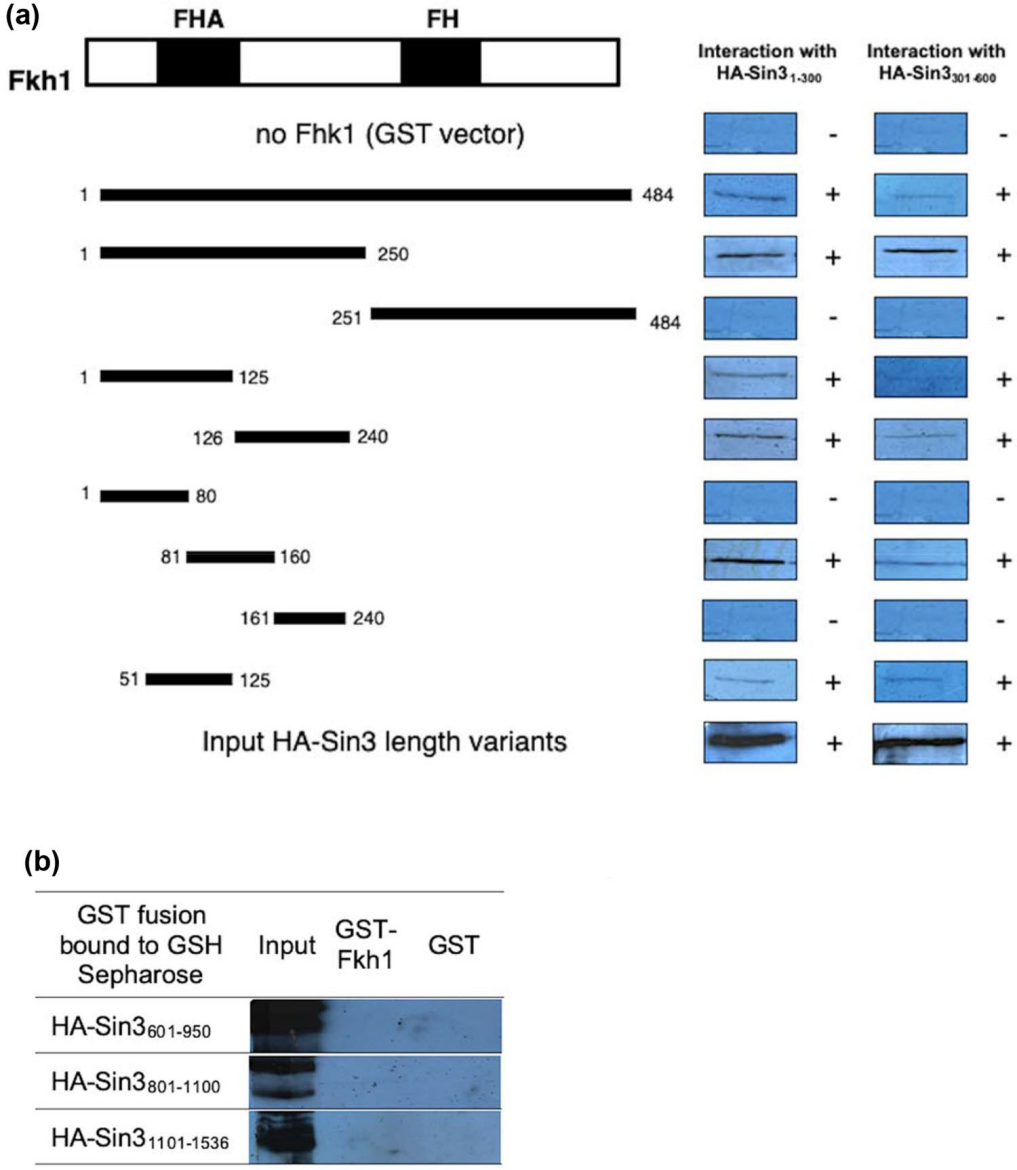
Physical map of Fkh1 domains interacting with Sin3. **a** Mapping of Fkh1 domains binding to PAH1 and PAH2 of Sin3. Length variants of Fkh1 were fused with GST, immobilized on GSH Sepharose and incubated with protein extracts from yeast. The following *GST-FKH1 E. coli* expression plasmids were used: pRAR2 (aa 1–484 of Fkh1), pRAR8 (aa 1–250), pRAR9 (aa 201–484), pRAR16 (aa 1–125), pRAR17 (aa 126–240), pRAR32 (aa 1–80), pRAR33 (aa 81–160), pRAR34 (aa 161–240) and pRAR73 (aa 51–125). HA-tagged Sin3 length variants were synthesized in *S. cerevisiae* using pCW83 (aa 1–300) and pYJ91 (aa 301–600). Input controls for both Sin3 length variants are shown at the bottom of Fig. (20% of protein used for the interaction assay). FH, forkhead domain; FHA, forkhead associated. **b** Full-length Fkh1 does not interact with the C-terminus of Sin3. Full-length Fkh1 was bacterially synthesized using pRAR2. C-terminal length variants of Sin3 (comprising PAH3 and PAH4) were synthesized in *S. cerevisiae* by use of pYJ90 (aa 601–950), pYJ89 (aa 801–1100) and pMP20 (aa 1101–1536). For input controls (shown in the left panel), 20% of protein used for the interaction assay was analyzed

As is apparent from Fig. 3a, the Forkhead Associated domain of Fkh1 is to a certain extent in charge of Sin3 recruitment. Interestingly, the two length variants of Fkh1 (aa 1–125 and aa 126–240) can bind to PAH1 and PAH2 of Sin3. To inspect the minimal core domain of Fkh1 in charge of Sin3 recruitment, we tested shorter Fkh1 length variants which revealed that aa 81–160 of Fkh1 are able to mediate Sin3 binding. More precise, 75 amino acids at the N-terminus (residues 51–125, comprising the FHA) turned out as the domain which is sufficient to interact with PAH1 and PAH2 of Sin3.

Vice versa, the physical map of interacting domains within Sin3 (Fig. 3a) revealed that amino acids 1–300 and 301–600 of Sin3 comprising its domains PAH1 and PAH2, respectively, are sufficient for interaction with Fkh1. No interaction was detected with constructs representing C-terminal sequences of Sin3 (Fig. 3b).

### In vivo interaction of length variants of Fkh1 and Sin3

Accordingly, to support the obtained in vitro results by GST pull-down assays, we used two-hybrid analyses as a suitable technique for validating interaction in vivo. Length variants of Sin3 comprising PAH1 (aa 1–300) and PAH2 (aa 301–888) were fused with the DNA-binding domain (BD) of Gal4. Length variants of Fkh1 which in vitro showed Sin3 binding were fused with the Gal4 transcriptional activation domain (AD). In vivo Sin3-Fkh1 interactions should reconstitute a functional Gal4 activator being able to stimulate expression of the *GAL2-ADE2* reporter gene of recipient strain PJ69-4A. As a negative control, empty vectors containing BD and AD were used.

As is apparent from the results summarized in Table 1 (original results presented in Fig. S1; supplementary file), expectedly no growth on adenine-free medium could be detected for the negative control. Conversely, co-transformation of BD-Sin3 (aa 1–300 and aa 301–888) with AD fused to Fkh1 length variants (aa 1–125 and aa 126–240) restored growth on adenine-free medium, which is consistent with results of our in vitro analysis. Of note, Fkh1_51–125_ minimal binding domain which has displayed in vitro interaction with PAH2 of Sin3 is able to show in vivo binding as well, proving the interaction between Fkh1 core domain (aa 51–125) and PAH2 of Sin3.

In contrast to in vitro results, BD fusions of Sin3 (aa 1–300, containing PAH1) in combination with AD fusions of Fkh1 (aa 81–160 and aa 51–125) were unable to mediate growth on medium lacking adenine. Presumably, formation of functional interaction domains in vivo is prevented with certain length variants due to failure of correct protein folding. In summary, use of the in vivo two-hybrid system confirmed that PAH2 domain of Sin3 exclusively interacts with Fkh1.

**Table 1:** Interaction domains of Sin3 and Fkh1 shown in vivo by two-hybrid assay

Fusion constructs	Growth of transformants on	
	- Leu-Trp	-Leu-Trp -Ade
AD-Fkh1_1–125_ /BD-Sin3_1–300_	+	+
AD-Fkh1_1–125_ /BD-Sin3_301–888_	+	+
AD-Fkh1_126–240_ /BD-Sin3_1–300_	+	+
AD-Fkh1_126–240_ /BD-Sin3_301–888_	+	+
AD-Fkh1_81–160_ /BD-Sin3_1–300_	+	−
AD-Fkh1_81–160_ /BD-Sin3_301–888_	+	+
AD-Fkh1_51–125_ /BD-Sin3_1-300_	+	−
AD-Fkh1_51–125_/BD-Sin3_301–888_	+	+
AD/BD	+	−

*GAL4*_AD_-*FKH1* fusion constructs were co-transformed with *GAL4*_BD_-*SIN3* fusions, encoding amino acids 1–300 and 301–888 of Sin3, respectively. Amino acid positions of Fkh1 fragments fused to Gal4_AD_ are indicated. For all transformations, strain PJ69-4A (*GAL2-ADE2 GAL7-lacZ*; James et al. 1996) was used as a recipient. As a qualitative evidence for in vivo interaction, the growth of transformants on selective medium lacking adenine was characterized after 48 h incubation. As a negative control, empty pGBD-C1 (BD) and pGAD-C1 (AD) vectors were used. Growth in the absence of adenine is possible when a functional Gal4 activator is reconstituted by Fkh1-Sin3 interaction in vivo

### Mutational analysis of Fkh1 binding domain with Sin3

**Fig. 4:**
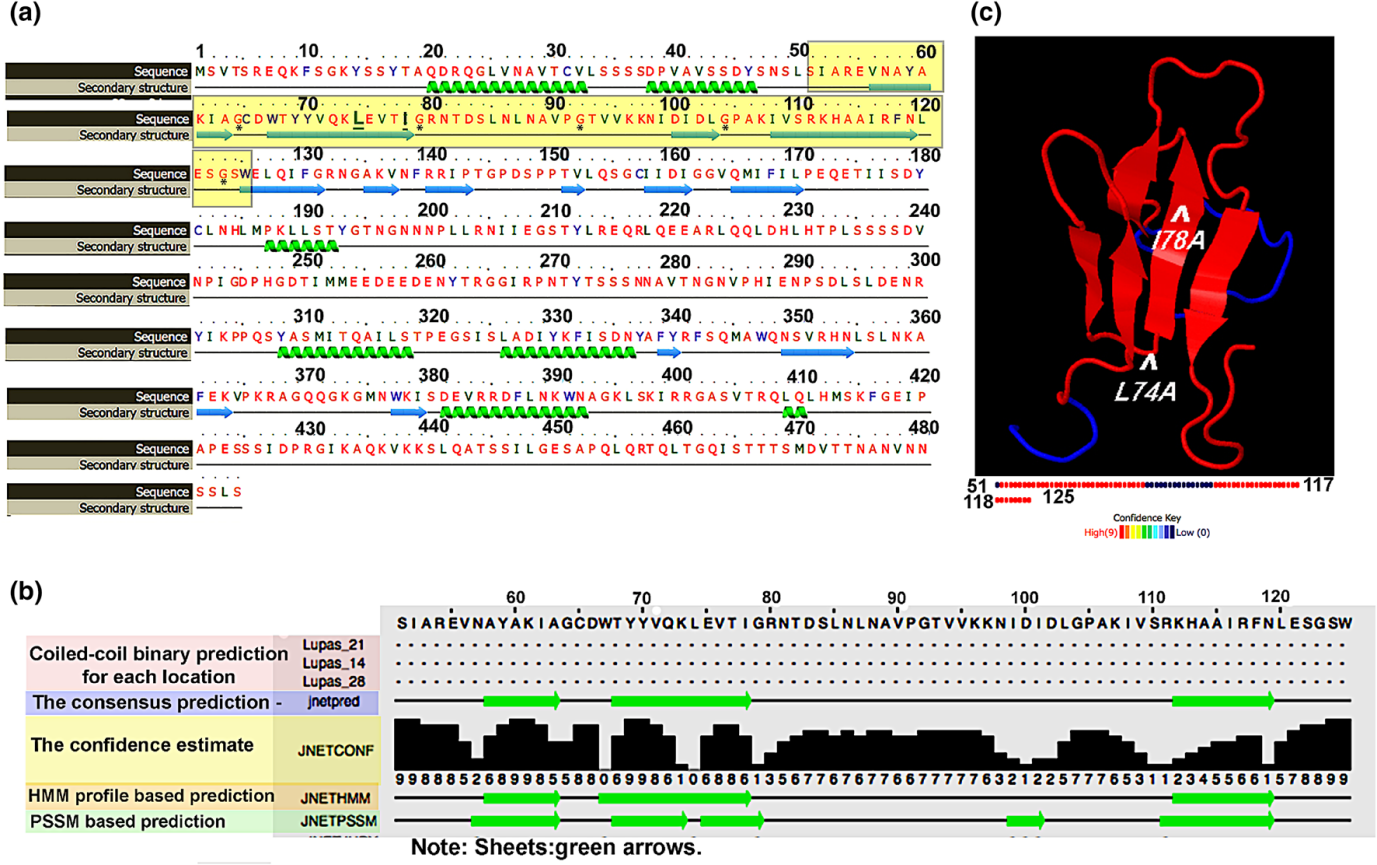
Prediction of the secondary and tertiary structure of Fkh1 core domain recruiting Sin3. **a** Prediction of the secondary structure of Fkh1 protein and the 75 aa sequence of Fkh1 subdomain recruiting Sin3 is highlighted in yellow that consists of five β-sheets. Within the Fkh1 core domain, the glycine (G) amino acid is denoted by an asterisk (*) to define the turns. Through the sequence, the amino acids that are substituted in the site-directed mutagenesis experiment are underlined (L74A, I78A). The prediction was done using Phyre2 software. **b** The Fkh1 core domain (aa 51–125) recruiting Sin3 was reanalyzed via various neural networks using Jpred 4 software (Cole et al. 2008). **c** The tertiary (3D) structure of the Fkh1 core domain (aa 51–125) was predicted by phyre 2 software and displayed using JSmol software (Hanson et al. 2013). The white arrows pointed to the positions L74A and I78A referred to as mutated amino acids (mentioned in the site-directed mutagenesis experiment). The 3D structure prediction scored 79% confidence represented in 59 out of 74 residues. Five antiparallel β-sheets were visualized by the JSmol model

Based on the methods of homology, the secondary structure of the full length of Fkh1 protein was predicted to consist of 15 β-sheets and 7 α-helix structures (Fig. 4a). The Fkh1 core domain recruiting Sin3 spanned between aa 51 to aa 125 forming 5 β-sheets (Fig. 4a; highlighted). The secondary structure of the Fkh1 core domain was reanalyzed to build a robust structure model by Jpred 4, the prediction is based on the homology with various neural networks such as; JNETPSSM based on the position-specific scoring matrices (PSSMs) for accurate prediction of DNA binding site, and JNetHMM based on hidden Markov models HMM (Fig. 4b). The JNETCONF indicated the confidence of predicting β-sheets structures, the confidence scale ranges between 0 and 9 (Fig. 4b). Moving to the tertiary structure (3D) of the Fkh1 core domain, structural motifs were characterized by high confidence structure (red color) of adjacent antiparallel β-sheets, which visualized in the JSmol of 3D model (Fig. 4c). Two β-hairpins were formed; one consists of three antiparallel β-sheets and another composed of two antiparallel β-sheets. The antiparallel β-sheets are linked by four loops or turns. These turns were remarked by glycine (G) residues (Fig. 4a).

**Fig. 5:**
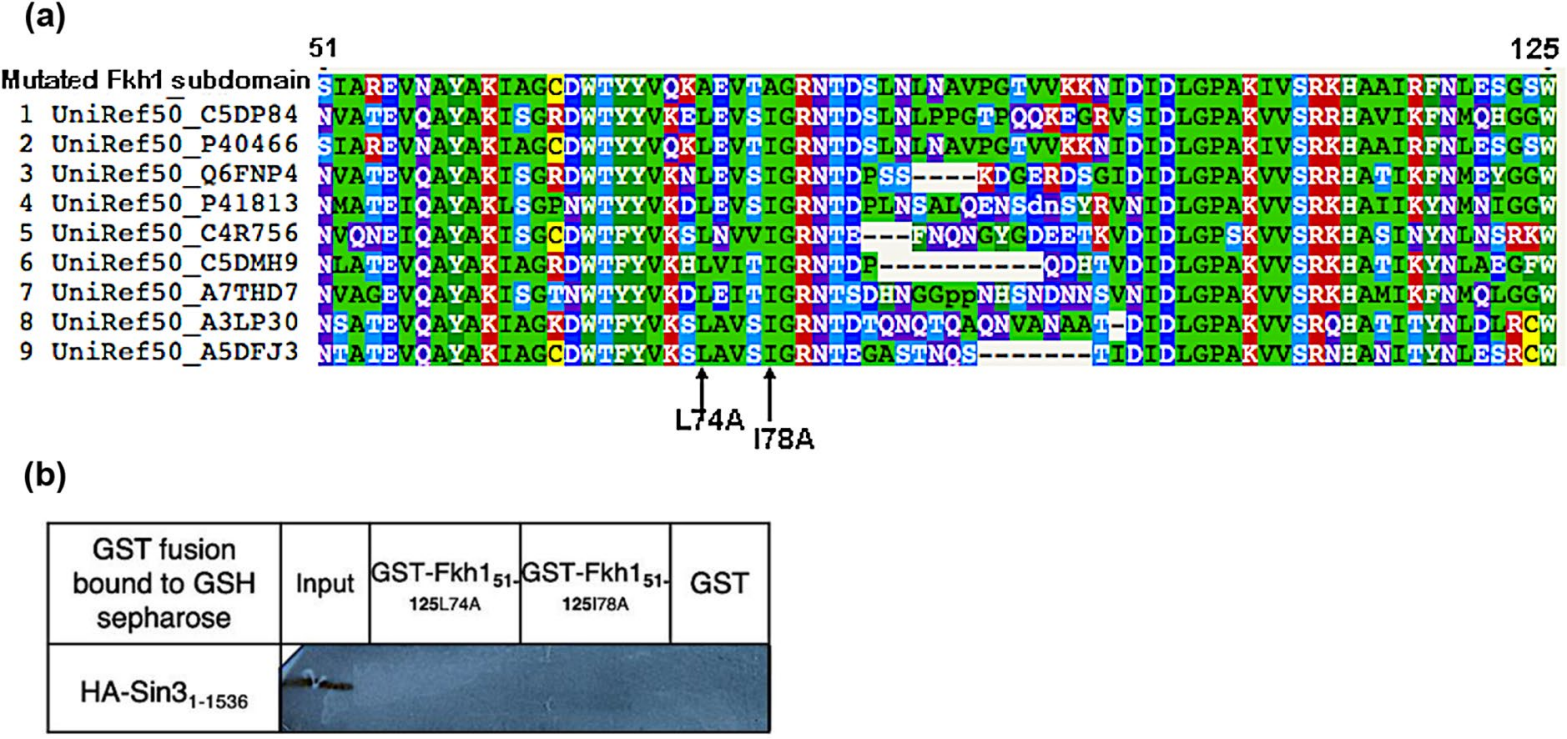
**a** The Mview of the multiple sequence alignment between a cluster of nine amino acid sequences from the UniRef knowledgebase that aligned with the mutated Fkh1 subdomain. The hydrophobic residues, leucine and isoleucine in positions 74 and 78 were found conserved. **b** In vitro interaction of GST-Fkh1 mutant variants and HA_3_-Sin3. GST-Fkh1_51–125_ comprising missense variants (plasmids pRAR89 and pRAR90) were comparatively analyzed for interaction with HA-tagged Sin3 expressed in *S. cerevisiae* (plasmid pCW117). GST-Fkh1 fusion proteins were released from GSH sepharose together with its partner by free GSH and subsequently separated by SDS-PAGE, followed by immunodetection using an anti-HA antibody. GST vector was used as a negative control. (20% of protein used for the interaction assay)

As aforementioned, the N-terminus of Fkh1 (residues 51–125) with its FHA domain is able to bind PAH2 of Sin3 in vitro and in vivo. Within the minimal identified domain of Fkh1, a short pattern of alternating hydrophobic amino acids which extends from aa 67–80 could be identified. To scrutinize the significance of these residues for interaction with Sin3 (and consequently for regulated expression of Fkh1 target genes), we implemented a site-directed mutagenesis at selected positions leading to the replacement of large hydrophobic amino acids to alanine [single mutations L74A and I78A; pointed out with arrows (Fig. 4c)]. To predict the stability of the structure and function after applying the site-directed mutagenesis using alanine (A) residues. We used PSI-BLAST to identify aligned protein sequences with the mutated Fkh1 minimal domain Then clustered sets of nine amino acid sequences from UniRef knowledgebase were selected for performing multiple sequence alignment in accordance with the mutated Fkh1 minimal domain (Fig. 5a).

The impact of alanine substitutions in Fkh1 domain (aa 51–125) on binding to Sin3 was subsequently investigated in vitro (by GST pull-down) and in vivo (two-hybrid system). To assay for in vitro interaction of Fkh1 missense variants with Sin3, GST-Fkh1 fusions (comprising aa 51–125) with mutational variants L74A and I78A were synthesized in *E. coli*, bound to GSH sepharose and incubated with a protein extract from *S. cerevisiae* containing HA_3_-Sin3. Figure 5b shows that both mutational variants (Fkh1_51–125_L74A and Fkh1_51–125_I78A) were completely defective for interaction with Sin3. In vivo binding of Fkh1 missense variants to Sin3 was again investigated by the two-hybrid system.

Thus, the AD-Fkh1_51–125_ fusion used above together with its mutational variants L74A and I78A were co-transformed into strain PJ69-4A, containing a plasmid encoding BD-Sin3_301–888_ (comprising PAH2). In contrast to the wild-type minimal binding domain (Fkh1_51–125_), Fkh1 variants L74A and I78A were unable to activate the *GAL2-ADE2* reporter gene, resulting in the failure of transformants to grow in the absence of adenine (summarized in Table 2, original results presented in Fig. S2; supplementary material). These findings unravel the pivotal role of Fkh1 amino acids 74 and 78 for binding to PAH2 of Sin3 corepressor.

**Table 2:** Mutational analysis of Fkh1-Sin3 interaction using two-hybrid constructs

Fusion constructs	Growth of transformants on	
	-Leu-Trp	-Leu-Trp-Ade
AD-Fkh1_51–125_/BD-Sin3_301–888_	+	+
AD-Fkh1_51–125 **L74A**_/BD-Sin3_301-888_	+	−
AD-Fkh1_51–125 **I78A**_/BD-Sin3_301–888_	+	−
AD/BD	+	−

The Gal4 DNA-binding domain (BD) was fused with a Sin3 fragment comprising PAH2 to give plasmid pJW50 (aa 301–888). Correspondingly, Gal4 transcriptional activation domain (AD) was fused with Fkh1_51-125_ wild-type and mutant variants to give pRAR79 (wild type), pRAR111 (L74A) and pRAR112 (I78A). As a negative control, empty vectors pGAD-C1 and pGBD-C1 were used. BD and AD pairs of fusion plasmids (selection markers: *TRP1* and *LEU2*, respectively) were co-transformed into strain PJ69-4A, containing a *GAL2-ADE2* fusion that allows growth in the absence of adenine when a functional Gal4 activator is reconstituted. Selection plates (-LT and -LTA; absence of leucine, tryptophan and adenine) were incubated for 48 h. Sequence of the mutagenized Fkh1 domain (residues 67–80): WTYYVQKLEVTIGR; amino acids that were replaced by alanine are underlined

### Fkh1-dependent recruitment of Sin3 corepressor to cell cycle-regulated genes

The functional relationship between Fkh1 and Sin3 corepressor led us to the hypothesis that Sin3 might be present at Fkh1 target genes. Chromatin immunoprecipitation assay (ChIP) was employed to monitor directly the occupancy of particular promoters by both regulatory proteins. A substantial body of data points to the significant role of Fkh1 in the regulation of the cell cycle (Kumar et al. 2000; Ostrow et al. 2014).

Fkh1 regulates the expression of the *CLB2* gene cluster (about 30 target genes) during the G2/M phase of the mitotic cell cycle (Mondeel et al. 2019). Noteworthy, an *fkh1* single deletion displays elevated *CLB2* transcription throughout the cell cycle (Hollenhorst et al. 2000). To address the regulatory function of Fkh1, we investigated whether Sin3 is directly bound at promoters of the *CLB2* cluster by performing ChIP assays with asynchronized cells (selected genes: *CLB2* and *SWI5*).

*CLB2* and *SWI5* are key players in cell cycle regulation (Veis et al. 2007). Accordingly, we explored the existence of Fkh1 and Sin3 each at promoters of both target genes (asynchronized cells) by ChIP analysis. This was achieved by constructing a strain (RAY4) encoding an epitope-tagged Fkh1-HA variant.

**Fig. 6:**
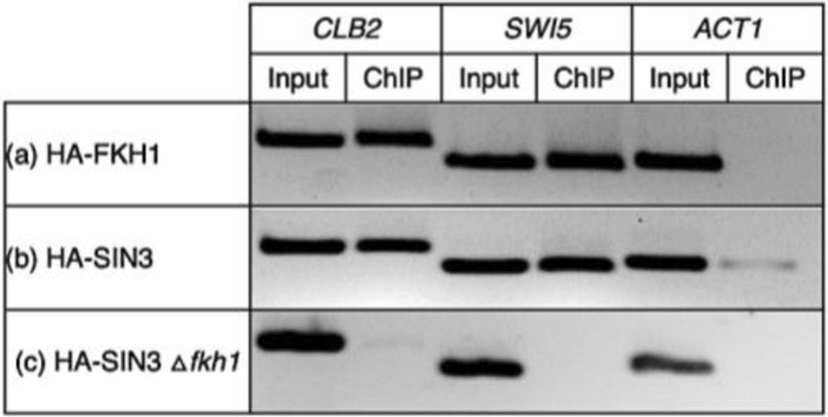
Fkh1-dependent Sin3 recruitment to promoters of cell cycle-regulated genes shown by chromatin immunoprecipitation. Strains RAY4 (contains a His-tagged variant of *FKH1* at its natural chromosomal position), FKH11 (contains a His-tagged variant of *SIN3* at its natural chromosomal position) and RAY5 (isogenic *fkh1* deletion mutant of FKH11) were grown to the exponential growth phase (non-synchronized cells). After shearing of chromatin, binding to His-Tag Dynabeads^®^ and elution, promoter fragments were analyzed by end-point PCR. **a** Recruitment of Fkh1 to *CLB2* and *SWI5* promoters. **b** Recruitment of Sin3 to *CLB2* and *SWI5* promoters. **c** Loss of Sin3 recruitment in the absence of Fkh1. DNA amplification was performed using specific primers for *CLB2* (− 880/− 580), *SWI5* (− 420/− 170) and *ACT1* (+ 841/ + 1165; negative control). PCR products were obtained after 29 amplification cycles and then separated by electrophoresis on a 2% agarose gel

A strain encoding an epitope-tagged variant of Sin3 (Sin3-HA; FKH11) has been previously described (Kliewe et al. 2020). Both HA-tagged Fkh1 and Sin3 proteins could be localized at *CLB2* and *SWI5* promoters in non-synchronized wild-type cells (Fig. 6a, b), inferring a functional interplay of both regulators for expression of the selected target genes. In vitro and in vivo experiments have shown Sin3 recruitment by Fkh1. Therefore, we further tested whether Sin3 recruitment depends on the function of the Fkh1 protein. An *fkh1* gene deletion was introduced into strain FKH11 which encodes an epitope-tagged Sin3 (to give strain RAY5; Sin3-HA *∆fkh1*) and then Sin3 recruitment to target genes was investigated again. As is apparent from Fig. 6c, Sin3 failed to bind to both promoters in the *∆fkh1* mutant strain although Sin3 was detected in the presence of an intact copy of Fkh1. Thus, Sin3 corepressor can be efficiently recruited to gene promoters when the Fkh1 protein is present.

### Fkh1 acts in concert with Sin3 mediating transcriptional repression in vivo

**Fig. 7:**
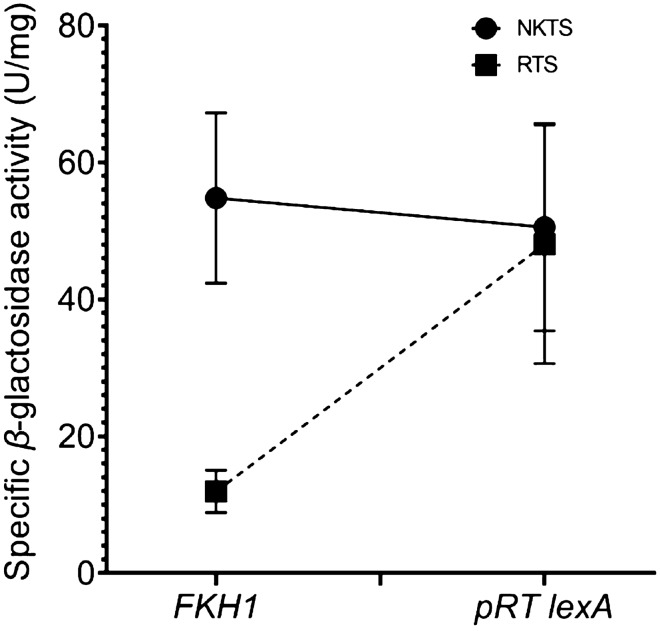
In vivo functional repression by Fkh1 recruited to a lexA_Op_-containing reporter gene. *S. cerevisiae* reporter strains RTS + lexA (integrated reporter gene [lexA_op_]_4_-*CYC1-lacZ*) and NKTS (reporter gene *CYC1-lacZ* without lexA_Op_) were transformed with effector plasmid pRAR28 (*lexA*_BD_-*FKH1*) and grown in SCD -Ura -Leu liquid medium to mid-log growth phase. Empty vector pRT-lexA served as a negative control. After cell harvesting, the specific β-galactosidase activity [µ/mg] was determined in crude extracts of the transformants

Although we here definitely confirmed the direct interaction between Fkh1 and Sin3, the in vivo significance of this result still remains open. According to previous findings, the assumption that Fkh1 may act as a repressor is obvious (Hollenhorst et al. 2000). To provide further evidence that Fkh1 indeed negatively affects gene expression and to quantify the presumed repression effect in vivo, an effector plasmid carrying the DNA-binding domain of the bacterial lexA repressor (lexA_BD_) together with the Fkh1 coding region was constructed. Plasmid pRAR28 (*lexA*_BD_-*FKH1*) together with the empty lexA plasmid pRT-lexA (negative control) was transformed into two strains containing integrated reporter genes (*CYC1-lacZ* without lexA-binding site; *CYC1-lacZ* with four upstream lexA-binding sites). As shown in Fig. 7 (original results presented in Table1 (section d); supplementary file), Fkh1 indeed conferred a significant reduction of the reporter gene expression when lexA-binding sites were present. Recruitment of Fkh1 to lexA-binding sites reduced the specific β-galactosidase activity almost fivefold. This result shows that Fkh1 mediates transcriptional repression when recruited to a promoter. Subsequent binding of corepressor Sin3 associated with HDACs will lead to inaccessible chromatin.

### Fkh1 protein targets Tup1 co‐repressor but not Cyc8

Previous studies have shown that repressor proteins may utilize more than a single corepressor complex to negatively affect transcription (Jäschke et al. 2011; Aref and Schüller 2020); . Investigation of the connection between Fkh1 and Cyc8/Tup1 complex revealed no current interaction is documented in STRING database (Fig. S3a; supplementary file) or even found in SGD database. Thus, we examined whether Fkh1 is also able to directly interact with corepressor Cyc8/Tup1. GST-Fkh1 immobilized on GSH sepharose could not associate in vitro with recombinant HA-tagged Cyc8 synthesized in *E. coli* (Fig. S3b; supplementary file). However, a corresponding experiment with GST-Fkh1 and *E. coli* extract containing HA-tagged Tup1 revealed a direct interaction between Fkh1 and Tup1 (Fig. S3c; supplementary file), indicating that the repressor function of Fkh1 may be redundantly executed by two separate corepressor complexes.

## Discussion

A primary goal of this study was to identify novel features of Fkh1 providing additional insights into its function by dissecting its pleiotropic ability for different HDAC regulators recruitment, mainly Sin3 and Tup1 in *S. cerevisiae*. *FKH1* (Forkhead Homologue One) was initially identified as a surrogate for *SIR1* (silent information regulator one) gene as it recovered mating ability in a *sir1* mutant (Hollenhorst et al. 2000). Fkh1 is a member of the conserved forkhead transcription factor family (Kumar et al. 2000) that has extensive molecular functions in multicellular eukaryotes (van der Horst and Burgering 2007). In yeast, Fkh1 (together with the related Fkh2) functions as a component of the *CLB2* gene cluster during the G2/M phase of the mitotic cell cycle (reviewed by Bähler 2005). Moreover, Fkh1 associates with a large number of functional genetic elements including origins of replication, centromeres and genes transcribed by RNA Pol III (Ostrow and Aparicio 2017).

Despite its negative role, no reports investigated in detail how Fkh1 executes HDACs recruitment function. Moreover, according to the PPI database of STRING, the interaction between Fkh1 and Sin3 is uncharacterized in *S. cerevisiae* so far. Although the putative homologs of Fkh1 and Sin3 scored co-expression, the confidence level was low (below 0.15). There was an initial study that highlighted Sin3 as an associated protein to Fkh1 through high-throughput mass spectrometric protein complex identification (Ho et al. 2002), In the meantime, no sufficient and confidential information was documented in the databases regarding this association yet. Thus, this study is the first one to characterize with significant impact and confidence the Fkh1/Sin3 interaction in *S. cerevisiae* with identifying the key binding domains within both partners.

In this work, we show by GST pull-down experiments the direct physical interaction between Fkh1 and pleiotropic corepressor Sin3. Forkhead proteins typically contain a DNA-binding domain together with a conserved Forkhead-associated (FHA) domain. While the DNA binding domain of Fkh1 turned out as inessential for Sin3 corepressor recruitment, we could demonstrate that aa 51–125 precisely covering its FHA domain are sufficient to bind PAH1 and PAH2 of Sin3 in vitro. Moreover, in vivo experiments indicated the inability of Fkh1 aa 81–160 and aa 51–125 length variants to bind PAH1 domain of Sin3 which suggests that PAH2 could be the genuine docking site playing role in stabilization of Fkh1/Sin3 interaction. Importantly, the FHA domain (at least of Fkh2) has been also described as a binding site for phosphorylated Ndd1 (dependent on protein kinases Cdk-Clb and Cdc5) which is indispensable for transcriptional activation of *CLB2* cluster genes (Reynolds et al. 2003; Darieva et al. 2006). Increased transcription of *CLB2* as a result of Fkh2-Ndd1 interaction triggered by Cdk-Clb is strong evidence for a positive autoregulatory loop. We speculate that FHA may have a dual function, being responsible for mediating contact to coactivators or corepressors, depending on the regulatory situation. When the Cdk-Clb activity is low (G1 phase of the cell cycle) and Ndd1 is dephosphorylated, Fkh proteins may instead recruit Sin3 which allows promoter access for various HDACs being associated with Sin3 (Rpd3, Hda1 and Hos1; Grigat et al. 2012).

Using a versatile in vivo repressor test system (initially described by Kadosh and Struhl 1997) we indeed could show that Fkh1 is able to repress transcription when fused to the DNA-binding domain of LexA. A lexA-Fkh1 fusion could repress transcription of a lexA_Op_-*CYC1-lacZ* reporter gene 4.6 fold in asynchronously growing cells. Similarly, Cti6 and Ume6 were able to repress transcription by factors of 5.6 and 7.0, respectively (Aref and Schüller 2020; Kadosh and Struhl 1997). As demonstrated for Ume6, we hypothesize that Fkh1 mediates repression through the recruitment of corepressors Sin3 and Tup1. Similarly, gene repression mediated by Opi1 (negative regulator of yeast phospholipid biosynthesis) was strongly alleviated in the absence of Sin3 and less effective in mutants *cyc8* and *tup1* (Wagner et al. 2001).

Previous work provided clear evidence that hydrophobic amino acids are of major importance for repressor-corepressor interactions (Sahu et al. 2008; Jäschke et al. 2011). This hypothesis is further supported in this work by the construction of Fkh1 variants mutated at selected hydrophobic residues (L74A and I78A) within the FHA domain, failing to bind Sin3 in vitro (assayed by GST pull-down) as well as in vivo (using a two-hybrid assay). The in-silico prediction displayed that the mutated amino acids are located within conserved hydrophobic patterns and within a β-sheet structure, which indicates its importance for protein stability and functionality. Particularly, the antiparallel orientation to form β-hairpins facilitates the protein–protein interaction (PPI) with robust quaternary structure three times more than the parallel β-sheet (Caudron and Jestin 2012; Wang et al. 2007; Zhao and Wu 2002).

**Fig. 8:**
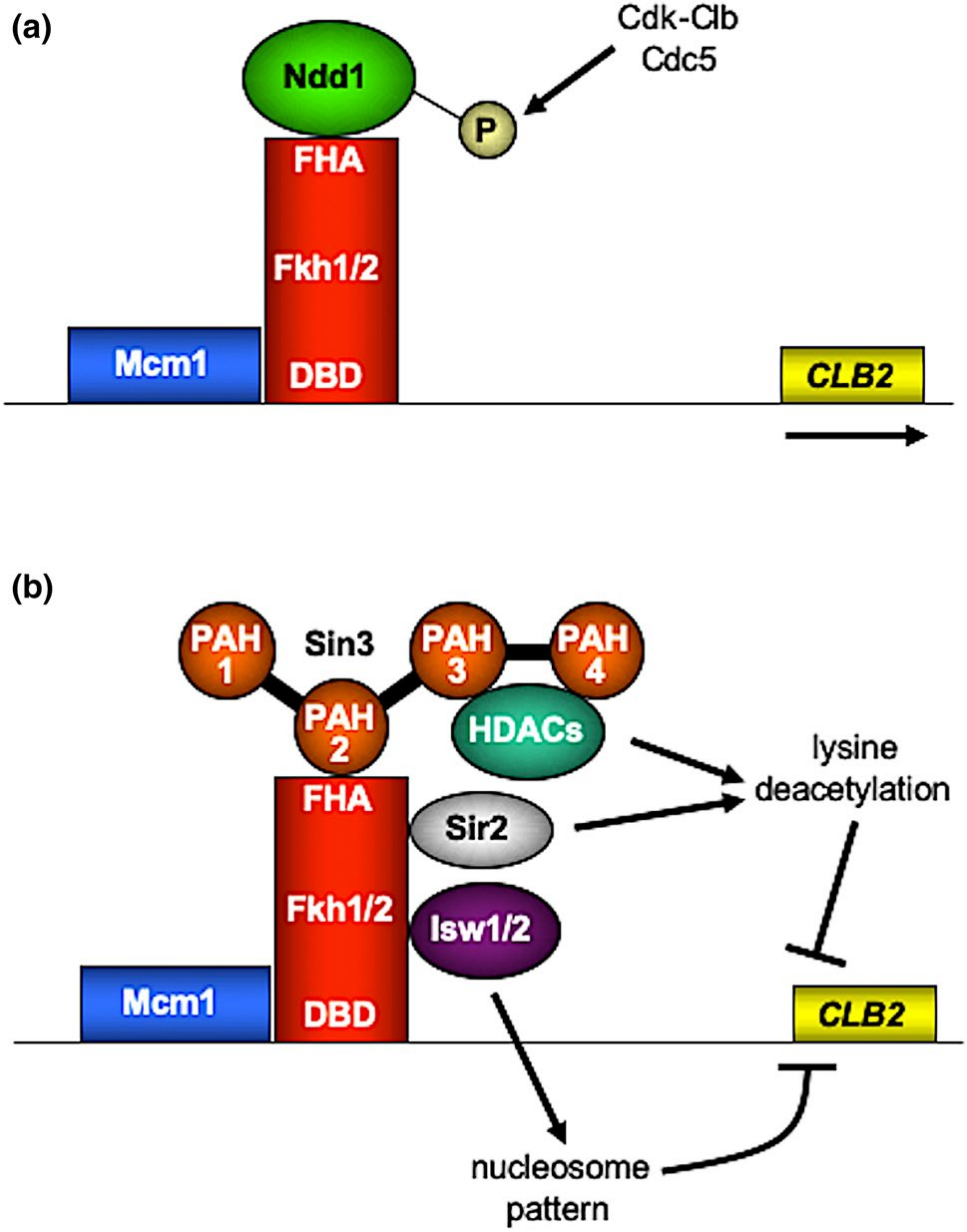
Schematic representation of Fkh-dependent regulation of *CLB2* cluster genes. *CLB2* is a cell cycle-regulated gene active from late S phase until G2/M transition. **a** Mcm1 and Fkh proteins bind to UAS elements upstream of genes of the *CLB2* cluster. Activation is triggered by phosphorylation of the essential coactivator Ndd1, requiring protein kinases Cdk-Clb and Cdc5 (Reynolds et al. 2003; Darieva et al. 2006). **b** Repression of the *CLB2* cluster in the G1 phase (no activity of Cdk-Clb) depends on Fkh proteins which recruit Sin3 corepressor (and possibly Tup1, not shown) through the interaction between FHA and PAH2. Sin3 then brings HDACs into action and thus prevents transcription of the respective genes. Repression is further supported by Fkh1-dependent recruitment of Sir2 histone deacetylase (Linke et al. 2013). In addition, Isw1 and Isw2 occupy *CLB2* promoter through Fkh1 and Fkh2 recruitment, respectively (Sherriff et al. 2007), initiating a repressive organization of chromatin. FHA, Forkhead-associated domain; PAH1-PAH4: paired amphipathic helices

To strengthen the in vivo significance of our results we also investigated chromatin occupancy of Fkh1 and Sin3 by ChIP. Although asynchronously growing cells were used, an association of Fkh1 and Sin3 with cell cycle-regulated promoters *SWI5* and *CLB2* could be clearly shown. Importantly, our ChIP analysis also confirmed that Sin3 is no longer existent at these promoters in the absence of Fkh1 (*∆fkh1* mutant), indicating that Sin3 recruitment is Fkh1-dependent. Previously, Veis et al. (2007) reported that recruiting Sin3 corepressor to *CLB2* promoter requires Fkh2 but not Fkh1. These authors also showed that promoter occupancy by Sin3 reached a peak at G1 but was substantially reduced in the S phase when *CLB2* activation started. Interestingly, a variation of Sin3 recruitment during G1 and G2/M phases coincides with dynamic nucleosome positioning and chromatin remodeling over the *CLB2* promoter region (Sherriff et al. 2007). Of note, FKh1 permanently occupies G2/M promoters (Hollenhorst et al. 2000; Kumar et al. 2000; Pic et al. 2000; Zhu et al. 2000). Notably, both Fkh1 and Fkh2 exist on the same promoters but occupy overlapped regions (Harbison et al. 2004; MacIsaac et al. 2006). The conversion of acetyllysine back to lysine may be executed simply by hydrolysis (HDACs Rpd3, Hda1, Hos1-3 in *S. cerevisiae*; reviewed by Yang and Seto 2008) or by a different mechanism requiring NAD (Sir2, Hst1-4; Landry et al. 2000). While our work emphasizes the importance of HDACs Rpd3, Hda1 and Hos1 bound to corepressors Sin3 and Tup1, Linke et al. (2013) additionally described the role of Sir2 deacetylase in controlling *CLB2* transcription. Besides covalent modification of histones, gene regulation of the *CLB2* cluster also requires reorganization of nucleosomes. While activators recruit chromatin remodeling complexes such as SWI/SNF or RSC to facilitate the formation of open chromatin, one additional aspect of repressor function may be promoting access of ISWI complexes to render chromatin less accessible. By studying the cleavage pattern of *CLB2* chromatin after treatment with micrococcal nuclease, Sherriff et al. (2007) could indeed show that the position of nucleosomes is dynamically shifted in the course of the cell cycle, requiring ATPases Isw1 and Isw2. Importantly, deletion of either *ISW1* or *ISW2* was able to suppress an otherwise lethal *ndd1* null mutation, emphasizing the importance of chromatin access for transcriptional activation in the absence of a genuine activator (Ndd1). Future studies should investigate whether Fkh1 (and/or Fkh2) directly interact with Isw1 and Sir2 under both repression and derepression conditions. As a conclusion, the findings of previous work and the results described here are summarized by the scenarios depicted in Fig. 8. Moreover, we launched for the first time an existence of direct interaction between Fkh1 and Tup1 validated by GST-Pull down. Such finding supports for gene transcription mechanisms controlled by one protein regulator that recruits multiple HDACs (Aref and Schüller 2020). Further investigation should be done in Tup1 respective pathways to explore potential functions of Fkh1, especially in the absence of any documented information regarding this interaction in the databases. Taken together, our results provide a prominent headway toward a full-fledged picture of Fkh1 roles with pleiotropic HDACs including cell cycle regulation.

## Supplementary Information

Below is the link to the electronic supplementary material.

Supplementary file1 (DOCX 5485 KB)
