# Supplementary material for: Forkhead transcription factor Fkh1: insights into functional regulatory domains crucial for recruitment of Sin3 histone deacetylase complex
Source: Curr Genet. 2021 Feb 26;67(3):487–99. doi: 10.1007/s00294-021-01158-3 (PMC8139909; doi:10.1007/s00294-021-01158-3)
Supplement: Supplementary file 1 — Supplementary file1 (DOCX 5485 KB) [file 294_2021_1158_MOESM1_ESM.docx]

**Supplementary File (Aref et al.)**

**Table 1:**

**a) Strains of *Saccharomyces cerevisiae* constructed and used by Aref et al.**

| **Strain** | **Genotype** |
| --- | --- |
| C13-ABY.S86 | *MAT*α *ura3 leu2 pra1 prb1 prc1 cps1* |
| PJ 69- 4A | *MAT*α *trp1-901 leu2-3,112 ura3-53 his3- 200 ∆gal4 ∆gal80 GAL2-ADE2 lys2::GAL1-HIS3 met2::GAL7-lacZ* |
| FKY11 | *MAT*α *ura3 leu2 pra1 prb1 prc1 cps1 SIN3-HA_3_::kanMX* |
| RAY4 | *MAT*α *ura3 leu2 pra1 prb1 prc1 cps1 FKH1-HA_3_::kanMX* |
| RAY5 | *MAT*α *ura3 leu2 pra1 prb1 prc1 cps1 SIN3-HA_3_::kanMX*  Δ*fkh1:: LEU2* |
| NKTS | *MAT*α *ura3 leu2 his3 trp1 CYC1-lacZ::URA3* |
| RTS + lexA | *MAT*α *ura3 leu2 his3 trp1 lexA_OP_-CYC1- lacZ::URA3* |

**b) Plasmids constructed and used by Aref et al.**

| **Plasmid** | **Genotype (Source)** |
| --- | --- |
| pCW117 | 2μm *URA3 MET25*_Pr_-HA_3_-*SIN3-CYC1*_Ter_ (full length); Wagner *et al.* 2001 |
| pSW11 | tet_Pr/Op_-HA_3_-*SIN3* (full length); Grigat *et al.* 2012 |
| pRAR110 | tet_Pr/Op_-HA_3_-*TUP1* (full length) |
| pFK77 | tet_Pr/Op_-HA_3_-*CYC8* (10 TPR Motifs, aa 1-398) |
| pCW83 | 2μm *URA3 MET25*_PRO_-HA_3_-*SIN3*_1-300_ *CYC1*_TER_; Jäschke *et al.* 2011 |
| pYJ91 | 2μm *URA3* MET25_PRO_-HA_3_-*SIN3*_301-600_ *CYC1*_TER_; Jäschke *et al.* 2011 |
| pYJ90 | 2μm *URA3* MET25_PRO_-HA_3_-*SIN3*_601-950_ *CYC1*_TER_; Jäschke *et al.* 2011 |
| pYJ89 | 2μm *URA3* MET25_PRO_-HA_3_-*SIN3*_801-1100_*CYC1*_TER_; Jäschke *et al.* 2011 |
| pMP20 | 2μm *URA3* MET25_PRO_-HA_3_-*SIN3*_1101-1536_*CYC1*_TER_; Jäschke *et al.* 2011 |
| pJW6 | 2μm *TRP1* ADH1_PRO_*-GAL4*_DBD_*-SIN3*_1-300_*-ADH1*_TER_; Wagner *et al.* 2001 |
| pJW50 | 2μm *TRP1* ADH1_PRO_*-GAL4*_DBD_*- SIN3*_301-888_*-ADH1*_TER_; Wagner *et al.* 2001 |
| pRAR2 | tac_Pr/Op_-GST-*FKH1* (aa 1-484, full length) |
| pRAR8 | tac_Pr/Op_-GST-*FKH1* (aa 1-250) |
| pRAR9 | tac_Pr/Op_-GST-*FKH1* (aa 251-484) |
| pRAR16 | tac_Pr/Op_-GST-*FKH1* (aa 1-125) |
| pRAR17 | tac_Pr/Op_-GST-*FKH1* (aa 126-240) |
| pRAR32 | tac_Pr/Op_-GST-*FKH1* (aa 1-80) |
| pRAR33 | tac_Pr/Op_-GST-*FKH1* (aa 81-160) |
| pRAR34 | tac_Pr/Op_-GST-*FKH1* (aa 161-240) |
| pRAR73 | tac_Pr/Op_-GST-*FKH1* (aa 51-125) |
| pRAR21 | 2μm *TRP1 ADH1*_PRO_-*GAL*4_TAD_-*FKH1*_(aa 1-125)_-*ADH*1_TER_ |
| pRAR22 | 2μm *TRP1 ADH1*_PRO_-*GAL*4_TAD_-*FKH1* _(aa 126-240)_-*ADH*1_TER_ |
| pRAR38 | 2μm *TRP1 ADH1*_PRO_-*GAL*4_TAD_-*FKH1* _(aa 81-160)_-*ADH*1_TER_ |
| pRAR79 | 2μm *TRP1 ADH1*_PRO_-*GAL*4_TAD_-*FKH1* _(aa 51-125)_-*ADH*1_TER_ |
| pRAR89 | tac_Pr/Op_-GST-*FKH1*_(aa 51-125)_ L74A, derived from pRAR73 |
| pRAR90 | tac_Pr/Op_-GST-*FKH1*_(aa 51-125)_ I78A, derived from pRAR73 |
| pRT-lexA | 2μm *LEU2 MET25*-l*exA*_DBD_-NLS |
| pRAR28 | 2μm *LEU2 MET25*-HA_3_-lexA_DBD_-NLS-*FKH1* (aa 1-484, full length) |
| pRAR111 | 2μm *TRP1 ADH*1_PRO_-*GAL*4_TAD_-*FKh1*_51-125_ [L74A]-*ADH*1_TER_ |
| pRAR112 | 2μm *TRP1 ADH*1_PRO_-*GAL*4_TAD_-*FKh1*_51-125_ [I78A]-*ADH*1_TER_ |
| pRAR107 | Δ*FKH1::LEU2* |

aa, amino acids; Pr, promoter; Op, operator; Ter, terminator; Mut, mutation; tetratricopeptide repeats (TPR)

**c) Table of oligonucleotides used by Aref et al.**

**(Construction of expression plasmids for GST-tagged Fkh1 (full length and length variants); PCR primers for *FKH1* strain modifications and *CLB2* and *SWI5* for ChIP analyses)**

| Name | Gene | Position | Sequence (5'-3') |  |  |
| --- | --- | --- | --- | --- | --- |
| FKH1 START *Bglll* | *FKH1* | +1 / +20 | gactagatctATGTCTGTTACCAGTAGGGA |  |  |
| FKH1 STOP *Xhol* | *FKH1* | +1455/+1436 | gactctcgagTCAACTCAGAGAGGAATTGT |  |  |
| FKH1 3R aa 250 *XhoI* | *FKH1* | +750/+731 | gactctcgagTCACGTGTCTCCATGTGGATCCC |  |  |
| FKH1 5F aa 250 *BamHl* | *FKH1* | +750/+769 | gactggatccATTATGATGGAAGAAGATGA |  |  |
| FKH1 5F aa 01 *BamHl* | *FKH1* | +1 / +20 | gactggatccATGTCTGTTACCAGTAGGGA |  |  |
| FKH1 3R aa 125 *Xhol* | *FKH1* | +375/+356 | gatcctcgagTCAACCATGATCCGCTCTCCAGGT |  |  |
| FKH1 5F aa 126 *BamHl* | *FKH1* | +378/+397 | gatcggatccGAATTACAGATATTCGGTAG |  |  |
| FKH1 3R aa 240 *Xhol* | *FKH1* | +720/+700 | gactctcgagTCAAACATCTGATGATGACGATA |  |  |
| FKH1 5F aa 251 *BamHI* | *FKH1* | +753/+772 | gactggatccATTATGATGGAAGAAGATGA |  |  |
| FKH1 3R aa 80 *Xhol* | *FKH1* | +240/+221 | gactctcgagTCACCTACCAATGGTAACTTCTA |  |  |
| FKH1 5F aa 81 *BamHl* | *FKH1* | +243/+262 | gactggatccAACACAGACAGCTTGAACTT |  |  |
| FKH1 3R aa 160 *Xhol* | *FKH1* | +480/+461 | gactctcgagTCAGTCTATGATACAACCAGACT |  |  |
| FKH1 5F aa 161 *BamHl* | *FKH1* | +483/+502 | gactggatccATAGGTGGTGTGCAAATGAT |  |  |
| FKH1 5F aa 251 *BglII* | *FKH1* | +753/+772 | gactagatctATTATGATGGAAGAAGATGA |  |  |
| FKH1 3R aa 240 *Hindlll* | *FKH1* | +720/+701 | gactaagcttTCAAACATCTGATGATGACGATA |  |  |
| FKH1 5F aa 51 *BamHl* | *FKH1* | +153/+172 | gatcggatccAGTATTGCCAGAGAGGTCAA |  |  |
| FKH1 3R aa 160 *Clal* | *FKH1* | +480/+461 | gactatcgatTCAGTCTATGATACAACCAGACT |  |  |
| FKH1 3R aa 125 *Clal* | *FKH1* | +375/+356 | gatcatcgatTCACCATGATCCGCTCTCCAGGT |  |  |
| FKH1 3R aa 240 *Clal* | *FKH1* | +720/+700 | gactatcgatTCAAACATCTGATGATGACGATA |  |  |
| FKH1 5F L74A | *FKH1* | +195/+244 | TGATTGGACGTATTATGTACAAAAA**GCA**GAAGTTACCATTGGTAGGAACA |  |  |
| FKH1 3R L74A | *FKH1* | +244/+195 | TGTTCCTACCAATGGTAACTTC**TGC**TTTTTGTACATAATACGTCCAATCA |  |  |
| FKH1 5F I78A | *FKH1* | +205/+254 | TATTATGTACAAAAATTAGAAGTTACC**GCT**GGTAGGAACACAGACAGCTT |  |  |
| FKH1 3R I78A | *FKH1* | +254/+205 | AAGCTGTCTGTGTTCCTACC**AGC**GGTAACTTCTAATTTTTGTACATAATA |  |  |
| FKH1 5F | *FKH1* | +1410/+1452 | GGACGTAACAACAAACGCAAACGTGAACAATTCCTCTCTGAGTtcccaccaccatcatcatcac |  |  |
| FKH1 3R | *FKH1* | +1689/+1647 | TTTTCTGGCGGTTTCCTTAATCTAACATTAAAATAAATAAACTactatagggagaccggcagat |  |  |
| FKH1 Ver | *FKH1* | +1201/+1220 | CGAGGCGCCTCTGTGACAAG |  |  |
| FKH1-5F-*EcoRl* | *FKH1* | -449/-430 | gactgaaTTCGAGATTTTCTAACCGCT |  |  |
| Fkh1-5F-*BamHl* | *FKH1* | -43/-62 | gactggatcCGCACAATTTACACACTATT |  |  |
| FKh1-3F-*Xbal* | *FKH1* | +2005/+2024 | gacttctaGACCCGTTAAGAAATATCTT | |  |
| FKh1-3F-*Hindlll* | *FKH1* | +1915/+1888 | gactaagctt CTGGAAGGGTTGGCGGTA | | |
| FKh1-Ver | *FKH1* | +1940/+1921 | ATATTCTCGTATATTCTGAC | | |
| FKH1-ORF | *FKH1* | +1172/+1191 | GGAATGCTGGTAAATTGAGT | | |
| CLB2 5F | *CLB2* | -880/-899 | GAGTGCATTAGCACAGTGGAA | | |
| CLB2 3R | *CLB2* | -580/-561 | CCCATGCTATGAGATGCTAG | | |
| SWI5 5F | *SWI5* | -420/-439 | AGAACATCTCTGTTCCAATTC | | |
| SWI5 3R | *SWI5* | -170/-151 | CGTGCCAATACATAAATAGTG | | |

Artificially inserted cleavage sequences for restriction enzymes are shown in **bold**; capital letters represent genuine gene-specific sequences.

**d) Gene repression in vivo by Fkh1 recruited to a lexA_Op_-contai­ning reporter gene.** *S. cerevisiae* reporter strains RTS+lexA (integrated reporter gene [lexA_op_]_4_-*CYC1-lacZ*) and NKTS (reporter gene *CYC1-lacZ* without lexA_Op_) were transformed with effector plasmid pRAR28 (*lexA*_BD_-*FKH1*) and grown in SCD-Ura-Leu liquid medium to mid log growth phase. Empty vector pRT-lexA served as a negative control. After cell harvesting, the specific β-galactosidase activity [U/mg] was determined in crude extracts of the transformants. Each experiment represents the mean value (in bold) of 4 independent strain cultivations and enzyme assays. ±*SD* standard deviation. The respective standard deviation is given in parenthesis.

| **Plasmids** | Specific β-galactosidase activity (U/mg) | | **Repression-Factor** |
| --- | --- | --- | --- |
|  | **NKTS**  (0x lexA) | **RTS**  (4x lexA) |  |
| pRAR28 | **55** (9) | **12** (2) | 4.6 |
| pRT | **51** (14) | **47** (19) | 1 |


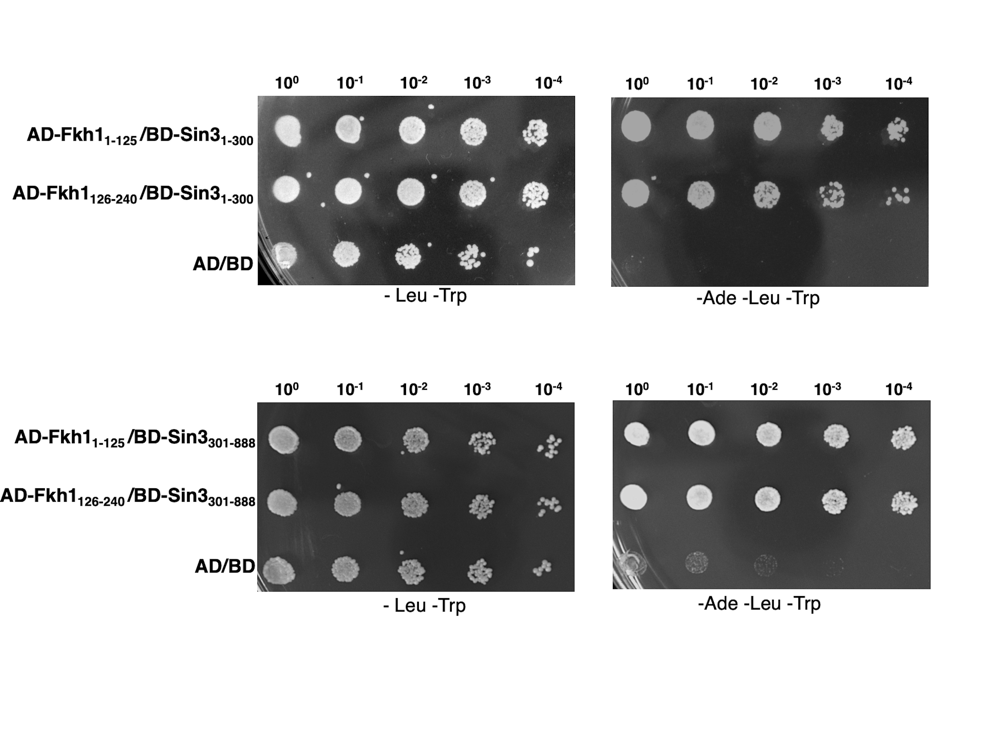


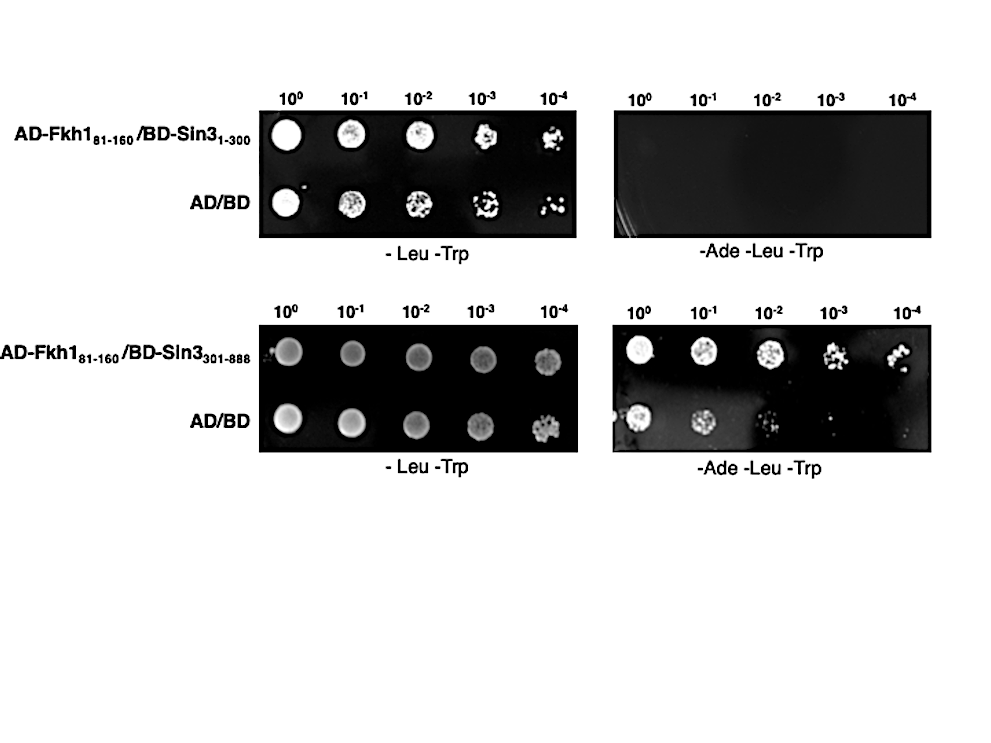


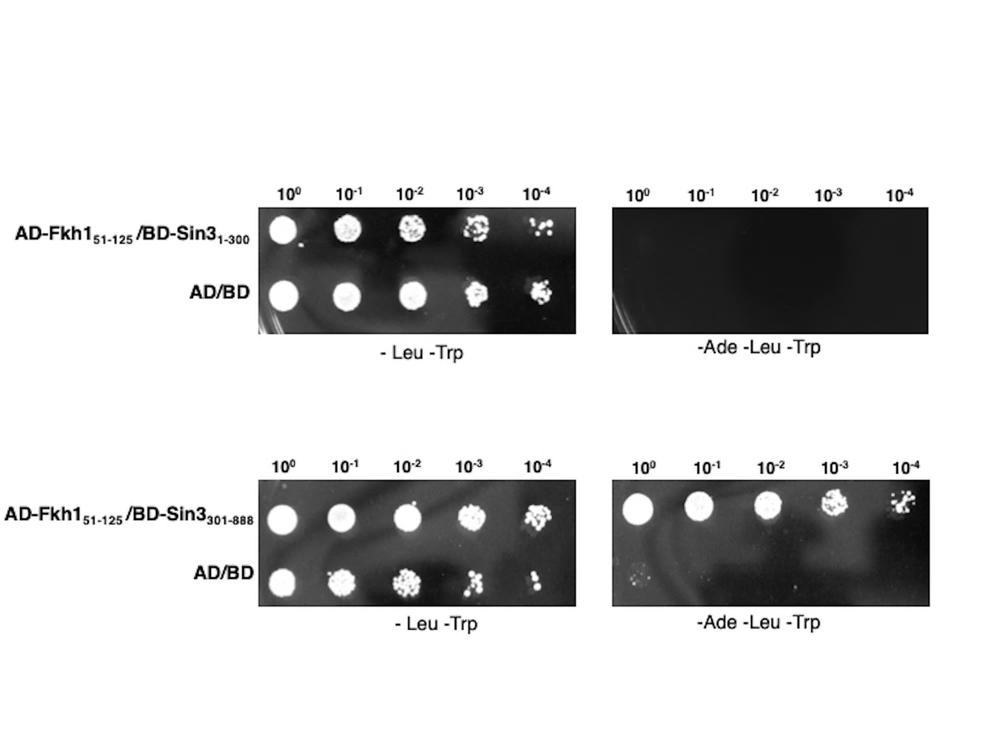


**Figure S1:** **Interaction domains of Sin3 and Fkh1 shown by two-hybrid assay**.

*GAL4*_AD_-*FKH1* fusion constructs were co-transformed with *GAL4*_BD_-*SIN3* fusions, encoding amino acids 1–300 and 301-888 of Sin3, respectively. Amino acid positions of Fkh1 fragments fused to Gal4_AD_ are indicated. For all transformations, strain PJ69-4A (*GAL2-ADE2 GAL7-lacZ*; James *et al*., 1996) was used as a recipient. As qualitative evidence for *in vivo* interaction, growth of transformants on selective medium lacking adenine was characterized after 48 h incubation. As a negative control, empty pGBD-C1 and pGAD-C1 vectors were used. Growth in the absence of adenine is possible when a functional Gal4 activator is reconstituted by Fkh1-Sin3 interaction *in vivo*.


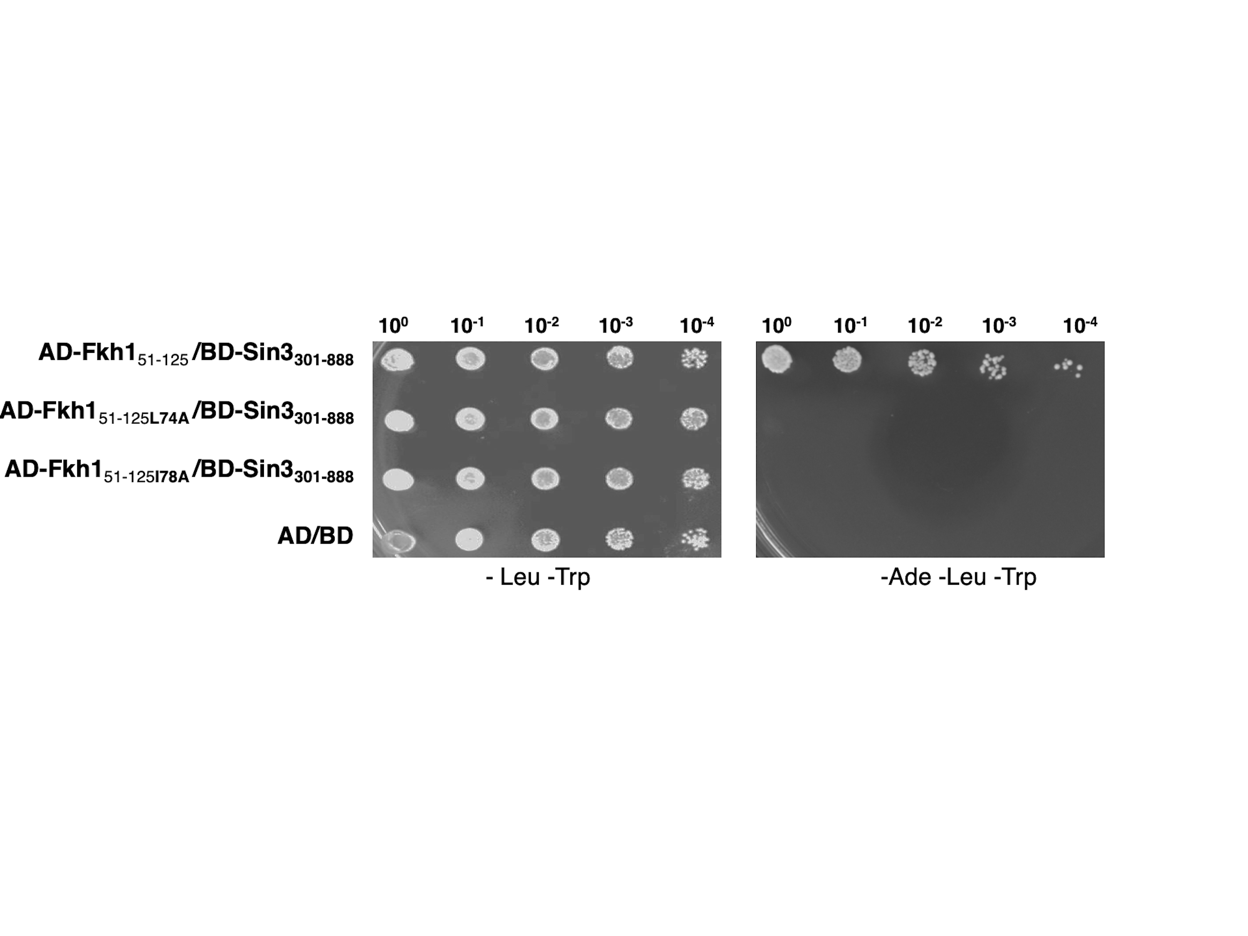


**Figure S2:** **Mutational analysis of Fkh1-Sin3 interaction using two hybrid constructs.**

The Gal4 DNA-binding domain (BD) was fused with a Sin3 fragment comprising PAH2 to give plasmid pJW50 (aa 301-888). Correspondingly, Gal4 transcriptional activation domain (AD) was fused with Fkh1_51-125_ wild-type and mutant variants to give pRAR79 (wild type), pRAR111 (L74A) and pRAR112 (I78A). As a negative control, empty vectors pGAD-C1 and pGBD-C1 were used. BD and AD pairs of fusion plasmids (selection markers: *TRP1* and *LEU2*, respectively) were co-transformed into strain PJ69-4A, containing a *GAL2-ADE2* fusion that allows growth in the absence of adenine when a functional Gal4 activator is reconstituted. Selection plates (-LT and -ALT; absence of adenine, leucine and tryptophan) were incubated for 48h.

**(a)**


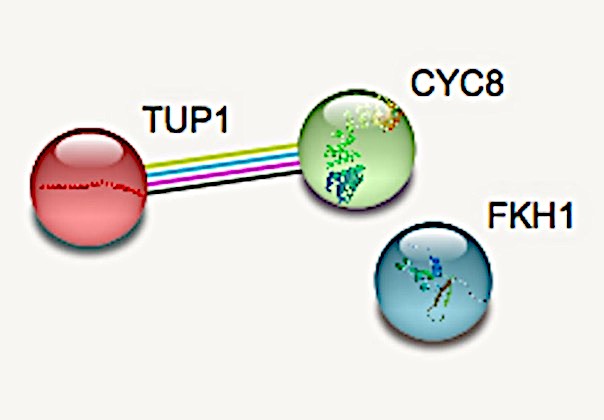


**(b)** Input Pull-down


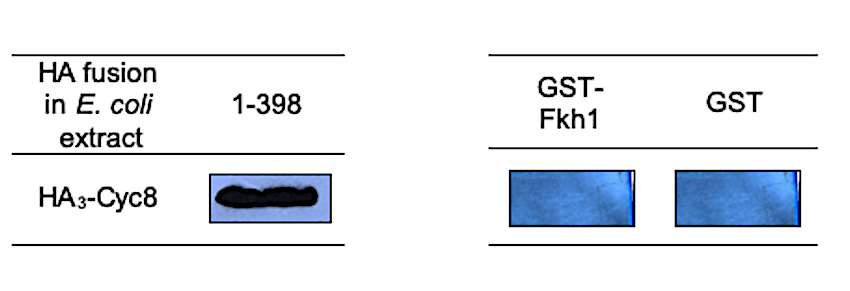


**(c)** Input Pull-down


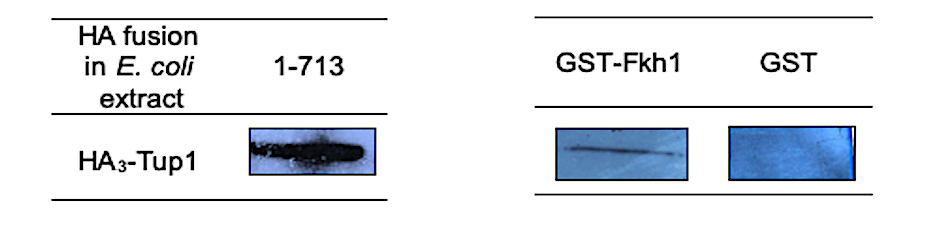


**Figure S3:** **In vitro interaction of Fkh1 with Cyc8 and Tup1 separately shown by affinity chromatography.**

**(a)** Fkh1 and Cyc8/Tup1 interaction network prediction in STRING database.

**(b)** GST-Fkh1 (full length fusion, pRAR2) was immobilized on GSH sepharose and incubated with protein extract from *E. coli* (strain BL21) containing HA_3_-Cyc8 (plasmid pFK77 encoding the N-terminal 10 TPR motifs of Cyc8).

**(c)** GST-Fkh1 (full length fusion, pRAR2) was immobilized on GSH sepharose and incubated with protein extract from *E. coli* (strain BL21) containing HA_3_-Tup1 (plasmid pFK76 encoding full-length Tup1).

Empty GST vector was used as a negative control. Extracts containing 75 µg of total protein were analyzed for the input control. To achieve comparable amounts of HA-Cyc8 and HA-Tup1 for the interaction assay, total protein was adjusted accordingly.
